# *LEA* Gene Expression Assessment in Advanced Mutant Rice Genotypes under Drought Stress

**DOI:** 10.1155/2019/8406036

**Published:** 2019-12-27

**Authors:** Zarifth Shafika Kamarudin, Mohd Rafii Yusop, Mohd Razi Ismail, Mahmud Tengku Muda Mohamed, Abdul Rahim Harun, Oladosu Yusuff, Usman Magaji, Arolu Fatai

**Affiliations:** ^1^Department of Crop Sciences, Faculty of Agriculture, Universiti Putra Malaysia, 43400 UPM Serdang, Selangor, Malaysia; ^2^Institute of Tropical Agriculture and Food Security, Universiti Putra Malaysia, 43400 UPM Serdang, Selangor, Malaysia; ^3^Agrotechnology and Biosciences Division, Malaysian Nuclear Agency, 43600 Kajang, Selangor, Malaysia

## Abstract

Late embryogenesis abundant (LEA) proteins are primarily found in plants stem, roots, and other organs and play significant roles in tolerance to several abiotic stresses. Plants synthesize a discrete set of LEA proteins in response to drought stress. In this study, the expression patterns of *LEA* genes were investigated in two advanced mutant rice genotypes subjected to the drought stress condition and different physiological traits including photosynthetic rate, leaf chlorophyll content, and photosystem II (PSII) photochemical efficiency (*Fv/Fm*) which were analyzed to confirm their drought tolerance. Five *LEA* genes (*OsLEA1*, *OsLEA2*, *OsLEA3*, *OsLEA4*, and *OsLEA5*) were used in the evaluation of rice genotypes and were significantly upregulated by more than 4-fold for MR219-4 and MR219-9. The upregulated genes by these two varieties showed high similarity with the drought-tolerant check variety, Aeron1. This indicates that these advanced mutant genotypes have better tolerance to drought stress. The changes in the expression level of *LEA* genes among the selected rice genotypes under drought stress were further confirmed. Hence, *LEA* genes could be served as a potential tool for drought tolerance determination in rice. MR219-4 and MR219-9 were found to be promising in breeding for drought tolerance as they offer better physiological adaptation to drought stress.

## 1. Introduction

The late embryogenesis abundant (LEA) proteins are primarily found in plants, covering a number of intrinsically unstructured proteins (IUPs). These small proteins ranging from 10 to 30 kDa are formed during the maturation drying process of embryo development [1, 2]. Most of LEA proteins are highly hydrophilic and belong to the hydrophilin family which is characterized by a high content of charged amino acid residues, as well as glycine and other small amino acids such as alanine, serine, or threonine [3]. On the other hand, many reports have also categorized LEA proteins by their thermal stability, nonglobular structure, and low complexity [4, 5]. Hence, LEA protein presence has been linked with cellular dehydration to tolerance, which may be induced by drying, saline conditions, or freezing.

Drought is a major factor limiting the optimal growth and development of plants. Stress occurring due to severe drought can be detrimental at all stages of plant development. Studies on the effect of drought stress on the leaf gas exchange have shown a reduction in net photosynthesis with a detrimental effect of drought stress on the diffusion of carbon dioxide in plant leaves [6, 7]. Similarly, reductions in the leaf chlorophyll content and chlorophyll fluorescence have been reported in cases of drought stress [8, 9]. Usually, under drought stress conditions, the mean value of photosystem II (PSII) photochemical efficiency (*Fv/Fm*) decreases and this can serve as a good indicator for measuring drought stress [10].

In response to drought stress, plants synthesize a discrete set of LEA proteins which are believed to prevent and/or repair stress-induced damage [3]. These proteins are synthesized in response to water deficiency during a period of dryness or water shortage at an important stage of plant development [11]. LEA proteins are abundant in higher plant embryos. These proteins develop in response to drought and increase when plants are under severe drought stress condition [1].

*LEA* genes have been identified in many plants [12]. At least seven groups of LEA proteins have been categorized based on the similarities of their deduced amino acid sequences. These *LEA* function in protein protection upon water deficit where different hydrophilins, including LEA proteins from groups 2, 3, and 4, function to prevent the inactivation of enzymes such as lactate dehydrogenase (LDH) or malate dehydrogenase (MDH) depending on the level of dehydration [13]. In plants, a number of reports indicate that overexpression of LEA proteins from various groups confers tolerance to plant exposed to water-deficit treatments [2, 3, 14]. In rice, overexpression of *OsLEA3* enhanced drought tolerance in the field response to water-deficit stress [11]. More significantly, it has been shown that the deficiency of one, two, or three members of *LEA4* proteins from *Arabidopsis thaliana* predispose is enough to cause water-deficit susceptibility [14], thus showing the importance of these proteins in plant adaptive response to stress condition.

This study was conceptualized to determine drought-tolerant genotypes of advanced mutant rice genotypes by analyzing the physiological traits and expression patterns of *LEA* genes in two advanced mutant rice genotypes (MR219-4 and MR219-9) in comparison with MR219 (local rice variety) and Aeron1 (drought-tolerant variety) in response to drought stress condition for the purpose of building a foundation for drought tolerance determination in rice.

## 2. Materials and Methods

### 2.1. Plant Materials

Four rice genotypes comprising two advanced mutant lines (MR219-4 and MR219-9), a local rice variety (MR219), and a drought-tolerant as a check variety (Aeron1) were used in this experiment. The advanced mutant lines were derived from a preliminary study on development of drought tolerance using induced mutation through ion beam irradiation [15]. The gene expression analysis was carried out at the Food Crop Molecular Laboratory, Institute of Tropical Agriculture and Food Security, Universiti Putra Malaysia (UPM).

### 2.2. Differential Drought Stress Treatment

The experiment was conducted as a split-plot randomized complete block design with four replications. Rice seedlings were grown under well-watered and drought stress (withheld irrigation for 7 consecutive days) treatments at four weeks after transplanting. The experiment was conducted at Ladang 15 (latitude 2°59′1^″^N, longitude 101°44′6^″^E), Faculty of Agriculture, UPM. Five tensiometers (Takemura DM 8, Japan) were used in this experiment to determine the soil moisture tension. After subjecting the plant to drought stress and well-watered condition as treatments, leaf samples were collected and immediately suspended in liquid nitrogen for subsequent analysis.

### 2.3. Physiological Responses

#### 2.3.1. Photosynthesis Rate

Photosynthetic rate was recorded in 10 selected plants that were represented of each treatment. The measurement was recorded on the fully expanded and exposed leaves (third or fourth leaf from the tip) by using a portable photosynthesis system (Li-6400xt, Li-cor Inc., Lincoln, NE, USA) in the morning (09:00-10:00 am).

#### 2.3.2. Leaf Chlorophyll Content

Leaf chlorophyll content was measured in 10 plants per treatment. Fresh leaf sample (0.2 g) was cut into 0.5 cm pieces to determine the contents of leaf chlorophyll following the method described by Ashraf et al. [16]. Leaf sample of each genotype was kept overnight at -10°C in 25 ml tube containing 80% acetone. The mixture was centrifuged at 12000 × g for 10 min, and the absorbance of the supernatant was read against the blank (ethanol) using a Shimadzu UV spectrophotometer. The maximum absorbance of chlorophyll a and b was measured at 645 and 663 nm. The contents of chlorophyll were calculated using the following formulas:

(1) $$\begin{array}{c} C_{\text{a}}=12.70\times A_{663}–2.69\times A_{645},\\ C_{\text{b}}=22.90\times A_{645}–4.68\times A_{663},\\ C_{\text{a}+\text{b}}=C_{\text{a}}+C_{\text{b}}=20.21\times A_{645}+8.02\times A_{663},\\ \text{Chlorophyll a content }\left(\text{mg }{\text{g}}^{-1}\text{ FW}\right)=\frac{25\times C_{\text{a}}}{0.2\times 100},\\ \text{Chlorophyll b content }\left(\text{mg }{\text{g}}^{-1}\text{ FW}\right)=\frac{25\times C_{\text{b}}}{0.2\times 100},\\ \text{Chlorophyll a}+\text{b content }\left(\text{mg }{\text{g}}^{-1}\text{ FW}\right)=\frac{25\times C_{\text{a}+\text{b}}}{0.2\times 100}. \end{array}$$

#### 2.3.3. PSII Photochemical Efficiency (*Fv/Fm*)

After exposing the leaves to darkness for 10 minutes, *Fv/Fm* on the leaf surface of fully expanded leaves at the top shoots was recorded using a Portable Fluorescence Spectrometer Handy PEA (Plant Efficiency Analyzer Meter; Hansatech Instruments, Norfolk, UK). Data were collected at between 10:00 and 11:00 am. The fully expanded leaves in 10 plants from each genotype/treatment were sampled for *Fv/Fm* determination.

### 2.4. Total RNA Extraction and cDNA Synthesis

Total RNA was extracted from each test sample using the Trizol method described by Chomczynski and Sacchi [17], where 1.5 ml (1500 *μ*l) Trizol reagent was added to 0.5 g tissue and was incubated for 5 minutes at room temperature. Then, 300 *μ*l of chloroform was added into the mixture, and this was agitated for 15 seconds, followed by incubation for 5 minutes at room temperature. The mixture was centrifuged at 12000 × g for 15 minutes at 8°C to obtain phase separation. The aqueous upper phase was transferred into a new tube, 750 *μ*l isopropanol was added, and the mixture was incubated for 10 minutes at room temperature. The isolated upper phase was further centrifuged at 18000 × g at 4°C for 15 minutes, and the supernatant was discarded. The pellets were washed with 70% ethanol prepared by diethyl pyrocarbonate (DEPC) water and vortexed gently. This was centrifuged at 13000 × g at 4°C for 10 minutes, and the supernatant was discarded. The pellets were air-dried for 5 minutes and dissolved in an appropriate amount of RNase-free water by pipetting up and down. The samples were heated to 60°C for 10 minutes. The extracted RNAs were stored at -80°C in RNase-free water. The RNA integrity was verified using a NanoDrop 1000 spectrophotometer (Thermo Scientific, Wilmington, DE, USA). RNA with a 260/280 with 1.8-2.0 ratio were selected and stored in -80°C until further analysis. The first-strand cDNA was synthesized by incubation on thermal cycler 4 *μ*l total RNA, 1 *μ*l Oligo (dT), 1 *μ*l dNTP, and 8 *μ*l RNase-free dH_2_O for 5 minutes at 65°C. The cDNA was immediately chilled on ice; then, 4 *μ*l 5x first-strand buffer, 1 *μ*l power M-MLV reverse transcriptase, and RNase inhibitor were added to make a total volume of 20 *μ*l. The reaction was run for 60 minutes at 50°C and subsequently at 70°C for 10 minutes. The cDNA samples were chilled on ice and stored at -20°C until further analysis.

### 2.5. Primer Design

A set of *LEA* genes was identified following the National Center for Biotechnology Information (NCBI) search of the 7 classified genes, i.e., *LEA_1*, *LEA_2*, *LEA_3*, *LEA_4*, *LEA_5*, *Dehydrin*, and *SMP*. Fifty-five primers ([Supplementary-material supplementary-material-1]) were selected from NCBI, and five target *LEA* gene-specific primers were confirmed after PCR amplification and gel electrophoresis. These five primers were used for the quantitative real-time PCR analysis for gene expression. Two reference genes, *Actin 11* (*ACT11*) and *Ubiquitin 5* (*UBQ5*), were used for normalization of the cDNA template quantity [18]. Primer details are shown in [Supplementary-material supplementary-material-1].

### 2.6. Polymerase Chain Reaction (PCR) and Gel Electrophoresis

The PCR amplification was performed in 15 *μ*l reaction consisting of 1 *μ*l 70 ng template cDNA, 1 *μ*l 10 *μ*M of each forward and reverse primer, 7.5 *μ*l DreamTaq Green PCR Master Mix (2x) (Thermo Scientific), and 4.5 *μ*l nuclease-free water using a thermocycler (T100TM, Bio-Rad). The PCR condition was attained using a conventional PCR program with the following profile: 94°C for 2 minutes followed by 34 cycles of 94°C for 30 seconds, 58°C for 30 seconds, then 72°C for 1 minute, and a final extension for 10 minutes at 72°C and subsequent rapid cooling to 4°C prior analysis. The LEA amplicons were separated by 1x TBE buffered electrophoresis on 3% *w*/*v* Metaphor agarose gel, stained with Midori green (1 *μ*l per 100 ml 1x TBE). The LEA amplicons were run at 90 volts for 1 hour and 15 minutes. A reference 50 base pair ladder (GeneDireX, Inc., Taiwan, ROC) suitable for use as molecular weight standards for Metaphor agarose gel electrophoresis was used to determine the LEA allele sizes of the amplified PCR products. The resultant bands were visualized under UV light and digitized using a Molecular Imager® imaging system (GelDocTM XR, Bio-Rad Laboratories Inc., USA).

### 2.7. Quantitative Real-Time PCR (qRT-PCR)

For the stress and well-watered condition, expression measurements were performed using quadruplicate biological replications. Quantitative PCR was performed in 20 *μ*l reactions using gene-specific primers ([Supplementary-material supplementary-material-1]). This consists of 3 *μ*l of cDNA template, 10 *μ*l Power 2x SYBR real-time PCR premixture (Fermentas, Shanghai, China), 0.4 *μ*l each of 10 *μ*M forward and reverse primers, and 6.2 *μ*l DEPC water. The reactions were subjected to 95°C for 2 minutes followed by 40 cycles of 95°C for 20 seconds, 60°C for 20 seconds, and 72°C for 60 seconds on the CFX96 Real-Time PCR system (Bio-Rad, UK). The primer specificity and the formation of primer dimers were monitored by dissociation curve analysis. The expression level of rice *ACT11* and *UBQ5* genes was used as internal standards for normalization of the treated cDNA template quantity ([Supplementary-material supplementary-material-1]). Controls (no cDNA template) were also included in the qPCR analysis.

### 2.8. Statistical Analysis

The data for all physiological parameters were subjected to standard statistical analysis such as analysis of variance (ANOVA) using SAS 9.4 software. The mean and coefficient of variation (CV) were calculated for each trait. The mean comparisons were performed using Tukey's Studentized Range (HSD) test. Data analysis of the *LEA* genes was performed using the software provided by Bio-Rad, UK. The comparative Ct (2^-*ΔΔ*Ct^) method was used to calculate the changes in gene expression as a relative fold difference between an experimental and calibrator sample. The genes that were up- or downregulated by more than 4-fold and with *p* ≤ 0.05 were considered to be differentially expressed.

## 3. Results

### 3.1. Physiological Parameters

The physiological parameters of the studied genotypes were assessed, and the results are presented in Table 1. Photosynthetic rate, leaf chlorophyll content, and *Fv/Fm* were reduced under drought stress condition. Plants treated with adequate water had the highest photosynthetic rate, leaf chlorophyll content, and *Fv/Fm* compared to the drought stress condition. Under drought stress condition, it was observed that MR219-9 showed the highest photosynthetic rate while MR219-4 showed the highest value in leaf chlorophyll content and *Fv/Fm* as compared to other rice genotypes.

### 3.2. Target and Reference Gene Validation

The efficiency, accuracy, reliability, and specificity of the *LEA* gene-specific primers and the reference genes were computed using a 3-5-fold serial dilution standard curve of cDNA template amplified on the thermal cycler real-time system. [Supplementary-material supplementary-material-1] shows the standard curve with the C_T_ plotted against the log of the starting quantity of template for each dilution. The equation for the regression line and the coefficient of determination (*R*^2^) values are shown in the graph ([Supplementary-material supplementary-material-1]). The calculated amplification efficiency was 101.9% for *OsLEA1*, 101.2% for *OsLEA2*, 98.3% for *OsLEA3*, 103.2% for *OsLEA4*, and 101.3% for *OsLEA5.* This indicates that the primers used are gene-specific and accurate for gene expression analysis.

### 3.3. Identification of the Target LEA Genes in the Candidate Rice Genotypes

A known-specific *LEA* gene primer of rice was used to examine the presence of the *LEA* genes in the studied rice genotypes. Five *LEA* genes from the 55 designed primers screened were consistently present in all the rice genotypes ([Supplementary-material supplementary-material-1]). PCR product was run to check the molecular weight (MW), only high molecular weight with clear bands was considered good, and smeared bands were considered poor in all rice samples. The MW was estimated using Image Lab software version 5.0 provided by Bio-Rad. These identified genes were considered for gene expression in the candidate drought-tolerant rice genotypes.

### 3.4. LEA Gene Amplification and Melt Curve Analysis in the Drought-Tolerant Rice Genotypes

**Figure 1:**
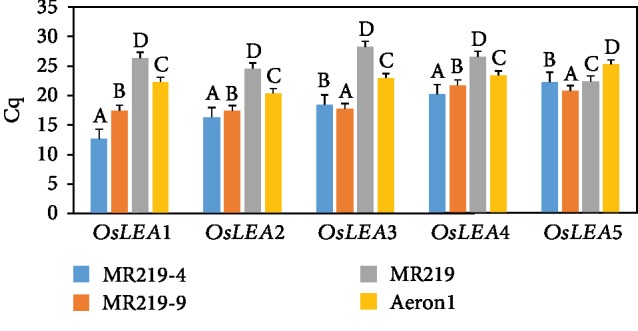
Amplification levels of candidate target *OsLEA1*, *OsLEA2*, *OsLEA3*, *OsLEA4*, and *OsLEA5* genes in the rice genotypes under drought stress condition; Cq values indicate mean of quadruplicate samples; vertical bars represent ±standard error; values within genotype with the different letter are significantly different based on comparison using the HSD test at *p* ≤ 0.05.

Quantitative real-time polymerase chain reaction (qRT-PCR) has been extensively used in several plant species as an accurate technique for gene expression analysis. In a real-time PCR assay, a positive reaction was detected by the accumulation of a fluorescent signal. Strong positive threshold cycle (Cq < 29) values were observed in all the studied drought-tolerant genotypes under drought stress condition (Figure 1). The entire five *LEA* transcripts were consistently abundant in the advanced mutant rice genotypes (MR219-4 and MR219-9) similar to Aeron1 (drought-tolerant variety). The analysis of variance for Cq values of all *OsLEA* genes is presented in [Supplementary-material supplementary-material-1].

Dissociation melt curve analysis was done to check for primer dimer and amplification of nonspecific products. [Supplementary-material supplementary-material-1] shows the melt curve of all the five LEA primers (*OsLEA1*, *OsLEA2*, *OsLEA3*, *OsLEA4*, and *OsLEA5*). Change in fluorescence with increasing temperature is measured as the melt curve analysis. As the temperature is increased, the two strands of the amplicon separate to form single-stranded DNA, causing the fluorescent intercalating dye to dissociate from the DNA and stop fluorescing.

### 3.5. Differential LEA Drought-Tolerant Gene Expression under Drought Stress and Well-Watered Condition

The analysis of variance for relative expression levels of the target *LEA* genes for each rice genotypes is presented in Table 2. The analysis of variance for each *LEA* genes was significantly different among the W, G, and G × W interaction.

**Figure 2:**
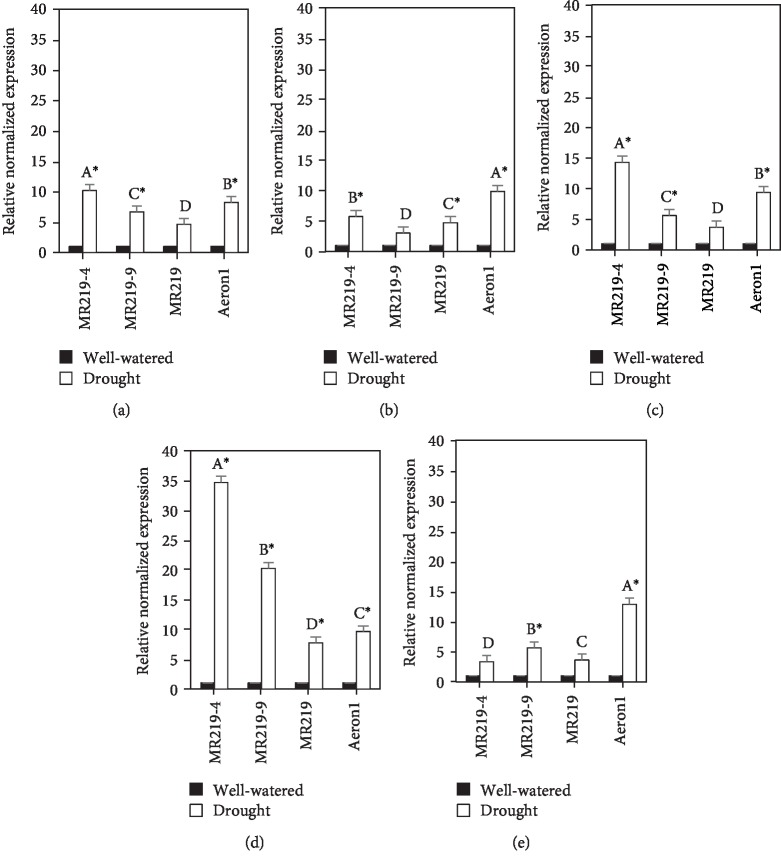
Relative expression levels of (a) *OsLEA1*, (b) *OsLEA2*, (c) *OsLEA3*, (d) *OsLEA4*, and (e) *OsLEA5* calibrated using *ACT11/UBQ5* reference genes in drought stress and control rice plants by relative quantitative real-time PCR; vertical bars represent ±standard error; values within genotype with the different letter are significantly different based on comparison using the HSD test at *p* ≤ 0.05; symbols (^∗^) indicate the *LEA* genes were significantly different by more than 4-fold change.

The *LEA* genes that were up- or downregulated by more than 4-fold and with *p* ≤ 0.05 were considered to be differentially expressed. MR219-9, MR219-4, MR219, and Aeron1 in this study showed differential expression: Os*LEA1*, Os*LEA2*, Os*LEA3*, Os*LEA4*, and Os*LEA5* were upregulated (Table 3, Figure 2). The upregulation of *LEA* genes in all the rice drought-tolerant genotypes indicates that *LEA* genes increased in the cell when exposed to drought stress.

## 4. Discussion

All rice genotypes showed decreased photosynthetic rate under drought stress condition unlike in rice genotypes given adequate water treatment (Table 1). The advanced mutant rice genotypes, MR219-4 and MR219-9, exhibit greater adaptation to drought. The photosynthetic apparatus that shows tolerance to desiccation of leaf water potential might be triggered for stomatal closure which in turn ensures optimal gas exchange in MR219-4 and MR219-9, thus reducing the transpiration rate. In the present study, MR219-4 showed the highest chlorophyll content among the rice genotypes under drought stress condition (Table 1), hence indicating that this genotype had higher photosynthetic efficiency compare to its parent, MR219, and other genotypes under study. On the other hand, it has been suggested that inhibition of PSII photochemistry could be associated with increased photoprotective energy dissipation or decreased photochemistry and photoinhibition associated with an overreduction of PSII which results in impairment in the functioning of the PSII system [19, 20]. The advanced mutant lines, MR219-4 and MR219-9, showed the ability to maintain higher *Fv*/*Fm* under water deficit indicating the higher efficiency of radiation use possibly in photochemistry and carbon assimilation. This result is supported by Guang-Cheng et al. [21]. The study further showed that MR219-4 had better performance in *Fv*/*Fm* compared to other rice genotypes indicating that MR219-4 is more tolerant to drought stress condition.

The amplification efficiency of 90–105% is the best indicator of a robust, reliable, and reproducible assay [22]. Low reaction efficiencies may be caused by poor primer design or by suboptimal reaction conditions. Reaction efficiencies > 100% may indicate pipetting error in the serial dilutions or coamplification of nonspecific products, such as primer dimers [23–25]. Gene expression analysis, especially on quantitative changes, has been simplified with the advent of qRT-PCR. Selection of optimal internal controls is very important to obtain reliable and accurate data in gene expression analysis using qRT-PCR [24, 26]. This is because normalization is imperative for gene expression analysis to avoid unnecessary errors in qRT-PCR analysis, and this is possible through the internal controls. The internal controls were selected from previous published works [18].

Drought stress is known to trigger changes in the transcription of *LEA* genes in drought-tolerant rice genotypes. The number of cycles required for the fluorescent signal to exceed background level which is inversely proportional to the amount of target nucleic acid in the sample is referred to as threshold cycle; the lower the Cq level, the greater the number of target transcripts in the sample [27]. In this study, all *LEA* genes significantly amplified early in the advanced mutant genotypes indicating abundant target nucleic acid in their respective leaf tissues under drought stress. Within the 7 consecutive drought stress exposure, these *LEA* genes were quickly induced to withstand the stress condition. Melt curve analysis is frequently used as a diagnostic tool for assessing qPCR amplicon length with intercalating dye qPCR assays. In this study, the curves showed clear specific peaks, indicating the primers are gene specific.

RNA isolated from leaf tissues collected at the vegetative stage was converted to cDNA and used for the quantitative RT-PCR analysis using *LEA* gene-specific primers in order to study the effect of change in the expression level in response to drought stress. From the present investigations, MR219-4 and MR219-9 advanced mutant rice genotypes could be considered drought-tolerant genotypes. This is because the increase in the expression of *LEA* genes often results in increased drought tolerance [11]. The expression of *LEA* in drought-tolerant genotypes is highly similar to Aeron1 genotype, indicating better protection in response to drought stress. This indicates that upon subjection to drought stress, the cellular drought response in the nucleus was activated. This leads to the enhanced expression of the *LEA* genes. Overexpression of *LEA* gene in rice was reported to have improved the drought resistance [18]. More significantly, it has been shown that the deficiency of one, two, or three members of LEA4 proteins from *Arabidopsis thaliana* is enough to cause water-deficit susceptibility [14], showing their relevance in the plant adaptive response to this stress condition.

## 5. Conclusions

The abundance of *LEA* genes was observed in the leaf tissues of the advanced mutant rice lines (MR219-4 and MR219-9) similar to the drought-tolerant check genotype after being subjected to drought stress. This indicates that drought-tolerant genotypes might offer better protection in response to drought stress. Different groups of *LEA* genes (*OsLEA1*, *OsLEA2*, *OsLEA3*, *OsLEA4*, and *OsLEA5*) were identified and expressed differently among the rice genotypes. A change in the expression level of the *LEA* gene among the genotypes under drought stress was further confirmed; hence, *LEA* could serve as a potential tool for drought tolerance determination in rice. The drought-tolerant advanced mutant lines are suggested for further drought-tolerant rice breeding program.

## Figures and Tables

**Table 1:** Mean values of photosynthetic rate, leaf chlorophyll content, and maximum quantum yield of PSII measured in the four studied rice genotypes under drought stress conditions.

Treatment/genotype	Physiological parameters					
	Photosynthetic rate (*μ*mol CO_2_ m^−2^ s^−1^)		Leaf chlorophyll content (mg g^−1^ FW)		PSII photochemical efficiency (*Fv*/*Fm*)	
	WW	DS	WW	DS	WW	DS
MR219-4	22.71^a^	12.32^b^	11.45^a^	5.44^a^	0.79^a^	0.62^a^
MR219-9	20.80^c^	13.43^a^	10.44^b^	2.82^b^	0.62^b^	0.51^b^
MR219	22.24^b^	12.28^b^	11.36^a^	2.45^c^	0.82^a^	0.44^d^
Aeron1	22.38^b^	12.32^b^	11.59^a^	1.89^d^	0.57^c^	0.46^c^
Mean	22.03	12.59	11.21	3.15	0.70	0.51
CV (%)	3.52	4.78	4.36	4.74	16.01	14.15

Note: means followed by the different letters within a column are significantly different from each other according to the HSD test at *p* ≤ 0.05; WW: well-watered; DS: drought stress; CV: coefficient of variation.

**Table 2:** Analysis of variance for relative expression levels of the five target *LEA* genes for each rice genotypes.

Source of variation	df	Mean square				
		*OsLEA1*	*OsLEA2*	*OsLEA3*	*OsLEA4*	*OsLEA5*
Replications (R)	3	0.05	0.02	0.01	0.05	0.03
Water treatments (W)	1	332.63^∗^	185.19^∗^	417.03^∗^	2329.20^∗^	227.54^∗^
R × W	3	0.05	0.02	0.01	0.05	0.03
Genotypes (G)	3	11.28^∗^	16.71^∗^	43.84^∗^	305.34^∗^	39.62^∗^
G × W	3	11.28^∗^	16.71^∗^	43.84^∗^	305.34^∗^	39.62^∗^
Error	18	0.04	0.06	0.04	0.03	0.02

^∗^Significant level at *p* ≤ 0.05.

**Table 3:** Relative expression patterns and *p* value of 5 *LEA* genes and *Actin*/*UBQ5* reference genes in two advanced mutant rice genotypes and two check varieties.

Gene	MR219-4	MR219-9	MR219	Aeron1
*OsLEA1*	UP 0.034	UP 0.021	UP 0.004	UP 0.021
*OsLEA2*	UP 0.000	No change 0.008	UP 0.040	UP 0.010
*OsLEA3*	UP 0.032	UP 0.022	No change 0.002	UP 0.030
*OsLEA4*	UP 0.045	UP 0.036	UP 0.023	UP 0.037
*OsLEA5*	No change 0.011	UP 0.009	No change 0.005	UP 0.023

UP = up regulation.

## Data Availability

The authors confirm that the data supporting the findings of this study are available within the article and its supplementary materials.

## Supplementary Materials

Supplementary Materials The supplementary files contain three tables and three figures as stated below. Table S1: show the list of total LEA gene screen for the study. Table S2: list of primers used for qRT-PCR amplification of LEA in rice. Table S3: analysis of variance for Cq values of candidate target *OsLEA1*, *OsLEA2*, *OsLEA3*, *OsLEA4*, and *OsLEA5* genes in the rice genotypes under drought stress condition. Figure S1: standard curve with the CT plotted against the log of the starting quantity of cDNA template for (A) *OsLEA1*, (B) *OsLEA2*, (C) *OsLEA3*, (D) *OsLEA4*, (E) *OsLEA5*, (F) *ACT11*, and (G) *UBQ5*. Figure S2: five *LEA* genes selected from the 55 screened designed primers in all rice genotypes with running on 3% metaphor agarose gel stained with Midori green. Figure S3: melting curve analysis of (A) *OsLEA1*, (B) *OsLEA2*, (C) *OsLEA3*, (D) *OsLEA4*, (E) *OsLEA5*, (F) *ACT11*, and (G) *UBQ5* genes.
