# Supplementary material for: LEA Gene Expression Assessment in Advanced Mutant Rice Genotypes under Drought Stress
Source: Int J Genomics. 2019 Dec 27;2019:8406036. doi: 10.1155/2019/8406036 (PMC7012254; doi:10.1155/2019/8406036)
Supplement: Supplementary Materials — The supplementary files contain three tables and three figures as stated below. Table S1: show the list of total LEA gene screen for the study. Table S2: list of primers used for qRT-PCR amplification of LEA in rice. Table S3: analysis of variance for Cq values of candidate target OsLEA1, OsLEA2, OsLEA3, OsLEA4, and OsLEA5 genes in the rice genotypes under drought stress condition. Figure S1: standard curve with the CT plotted against the log of the starting quantity of cDNA template for (A) OsLEA1, (B) OsLEA2, (C) OsLEA3, (D) OsLEA4, (E) OsLEA5, (F) ACT11, and (G) UBQ5. Figure S2: five LEA genes selected from the 55 screened designed primers in all rice genotypes with running on 3% metaphor agarose gel stained with Midori green. Figure S3: melting curve analysis of (A) OsLEA1, (B) OsLEA2, (C) OsLEA3, (D) OsLEA4, (E) OsLEA5, (F) ACT11, and (G) UBQ5 genes. [file 8406036.f1.pdf]

Supplementary Materials:

Table S1. List of total *LEA* genes available in National Center of Biotechnology Information (NCBI) (Access online at 18th February 2016)

No.	Gene name	Chromosome	Gene ID	NCBI file
1	*Os04g0589800*	4	4336815	LEA_1
2	*Os06g0110200*	6	4339887	LEA_1
3	*Os06g0324400*	6	4340902	LEA_1
4	*Os08g0327700*	8	4345287	LEA_1
5	*Os04g0685300*	4	4337461	LEA_2
6	*Os04g0416700*	4	4335811	LEA_2
7	*Os11g0120000*	11	9266873	LEA_2
8	*Os12g0159600*	12	4351561	LEA_2
9	*Os12g0159000*	12	4351557	LEA_2
10	*Os12g0119800*	12	4351350	LEA_2
11	*Os11g0130400*	11	4349673	LEA_2
12	*Os09g0267400*	9	4346564	LEA_2
13	*Os08g0494000*	8	4345917	LEA_2
14	*Os08g0324200*	8	4345274	LEA_2
15	*Os08g0102700*	8	4344425	LEA_2
16	*Os07g0531500*	7	4343454	LEA_2
17	*Os05g0584200*	5	4339745	LEA_2
18	*Os05g0526700*	5	4339385	LEA_2
19	*Os04g0685400*	4	4337462	LEA_2
20	*Os04g677300*	4	4337404	LEA_2
21	*Os03g0843300*	3	4334748	LEA_2
22	*Os03g0836300*	3	4334704	LEA_2
23	*Os03g0696000*	3	4333805	LEA_2
24	*Os03g0262700*	3	4332329	LEA_2
25	*Os02g0538700*	2	4329582	LEA_2
26	*Os01g0736500*	1	4327871	LEA_2
27	*Os01g0812100*	1	4327363	LEA_2
28	*Os01g0225600*	1	4326740	LEA_2
29	*Os01g0624400*	1	4326681	LEA_2
30	*Os10g0547200*	10	4349292	LEA_2
31	*Os08g0163600*	8	4344740	LEA_2
32	*Os07g0524200*	7	4343421	LEA_2
33	*Os07g0206800*	7	4342691	LEA_2
34	*Os05g0584300*	5	4339746	LEA_2
35	*Os02g0667600*	2	4330258	LEA_2
36	*Os02g0666600*	2	4330251	LEA_2
37	*Os07g0272300*	7	4342898	LEA_2
38	*Os06g0119900*	6	4339946	LEA_2
39	*Os02g0100600*	2	4327974	LEA_2
40	*Os01g0864500*	1	4324791	LEA_2
41	*Os02g0564600*	2	9267147	LEA_3
42	*Os01g0314800*	1	4327292	LEA_3
43	*Os05g0362600*	5	4338544	LEA_3
44	*Os03g0400700*	3	4333058	LEA_3
45	*Os03g0322900*	3	4332688	LEA_4

Table S1. Continued

No.	Gene name	Chromosome	Gene ID	NCBI file
46	*Os05g0349800*	5	4338499	LEA_5
47	*Os01g0702500*	1	4326935	LEA_5
48	*Os02g0669100*	2	4330265	Dehydrin
49	*Os11g0451700*	11	4350448	Dehydrin
50	*Os11g0454200*	11	4350453	Dehydrin
51	*Os11g0454000*	11	4350452	Dehydrin
52	*Os11g0453900*	11	4350451	Dehydrin
53	*Os06g0341300*	6	4340952	SMP
54	*Os03g0747400*	3	4334097	SMP
55	*Os03g0159600*	3	4331691	SMP

Table S2. List of primers used for qRT-PCR amplification of LEA in rice

Gene ID*	Gene name	Gene symbol	Amplicon length (bp)	Group	Primer sequence	E
4335811	*Os04g0416700*	*OsLEA1*	101	LEA_2	F: 5'-CTCCCTGTACGTCGTGAACA-3'<br>R: 5'-GCTTCTGTGTGTGCGTGTAG-3'	1.02
4351561	*Os12g0159600*	*OsLEA2*	132	LEA_2	F: 5'-TAGACCTCGACGTGGCAGTA-3'<br>R: 5'-CTCCAGGTCAAGATCGACGG-3'	1.01
4326681	*Os01g0624400*	*OsLEA3*	80	LEA_2	F: 5'-TAATTGTTAGCCGGCGCATC-3'<br>R: 5'-GTCCTTGATCAGCGACACGA-3'	0.98
4338544	*Os05g0362600*	*OsLEA4*	114	LEA_3	F: 5'-AGCTGTGTTGCCCTGCT-3'<br>R: 5'-AATCCTCTCCTGCACCG-3'	1.03
4331691	*Os03g0159600*	*OsLEA5*	109	SMP	F: 5'-ACAAGGACGCCGTGACGAT-3'<br>R: 5'-TCCCGGAGCTTGATCCTGT-3'	1.01
AK100267	*Actin 11*	*ACT11*	67	Reference gene	F: 5'-GGAACTGGTATGGTCAAGGC-3'<br>R: 5'-AGTCTCATGGATACCCGCAG-3'	1.04
AK061988	*Ubiquitin 5*	*UBQ5*	69	Reference gene	F: 5'-ACCACTTCGACCGCCACTACT-3'<br>R: 5'-ACGCCTAAGCCTGCTGGTT-3'	1.19

*Full-length cDNA gene ID; F: Forward primer sequences on full-length cDNA sequence; R: reverse primer sequences on full-length cDNA sequence; E, Amplification effieciency

Table S3. Analysis of variance for Cq values of candidate target *OsLEA1*, *OsLEA2*, *OsLEA3*, *OsLEA4* and *OsLEA5* genes in the rice genotypes under drought stress condition.

**Source of variation**	**df**	**Mean square**				
		OsLEA1	***OsLEA2***	***OsLEA3***	***OsLEA4***	***OsLEA5***
Replications	3	0.00042	0.000023	0.00002	0.00028	0.00010
Genotypes	3	142.267*	54.775*	94.583*	29.643*	14.198*
Error	9	0.00013	0.00010	0.00007	0.00014	0.00006

*Significant level at $p \leq 0.05$

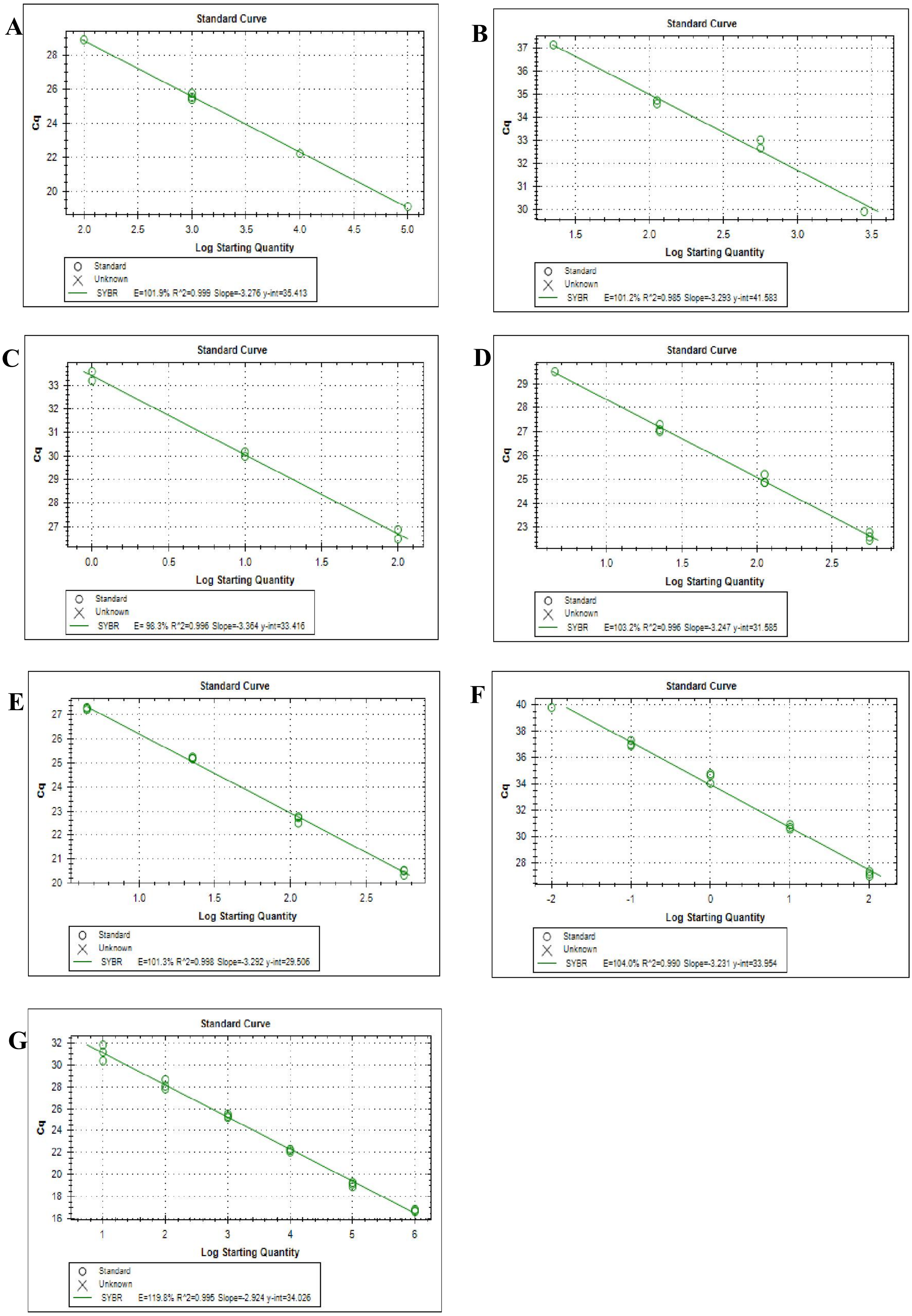

Figure S1. Standard curve with the C_T plotted against the log of the starting quantity of cDNA template for (A) *OsLEA1*; (B) *OsLEA2*; (C) *OsLEA3*; (D) *OsLEA4*; (E) *OsLEA5*; (F) *ACT11*; (G) *UBQ5*.

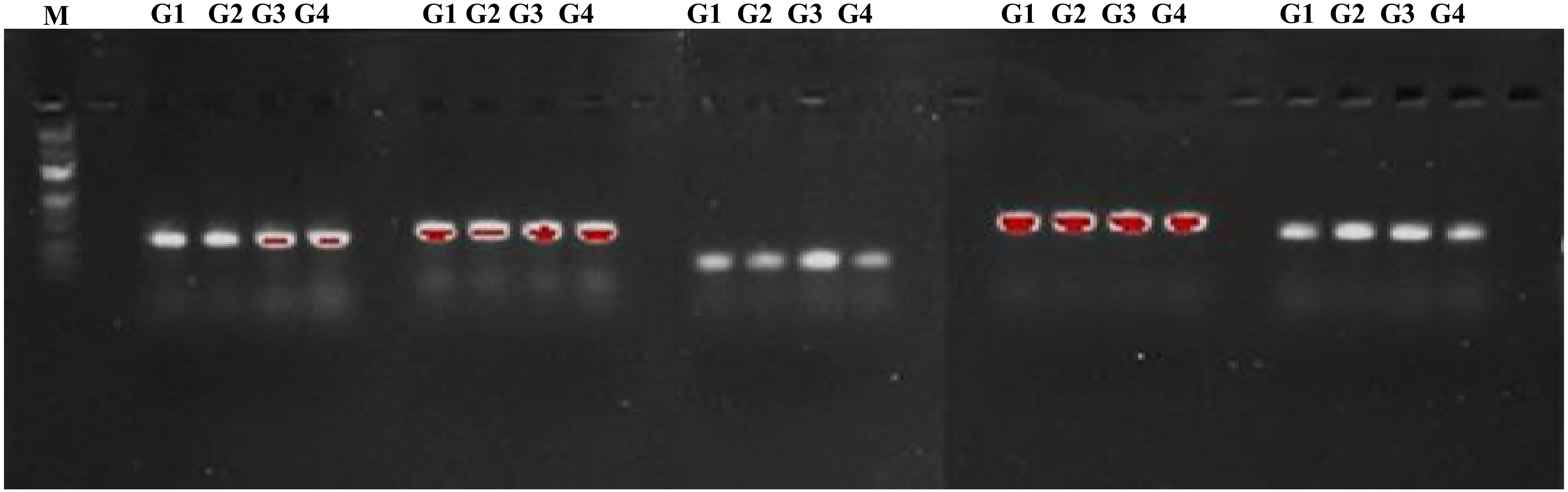

Figure S2. Five LEA (*OsLEA1, OsLEA2, OsLEA3, OsLEA4, OsLEA5)* genes selected from the 55 screened designed primers in all rice genotypes with running on 3% metaphor agarose gel stained with midori green (M=50 bp ladder), G1=MR219-4; G2=MR219-9; G3=MR219; G4=Aeron1

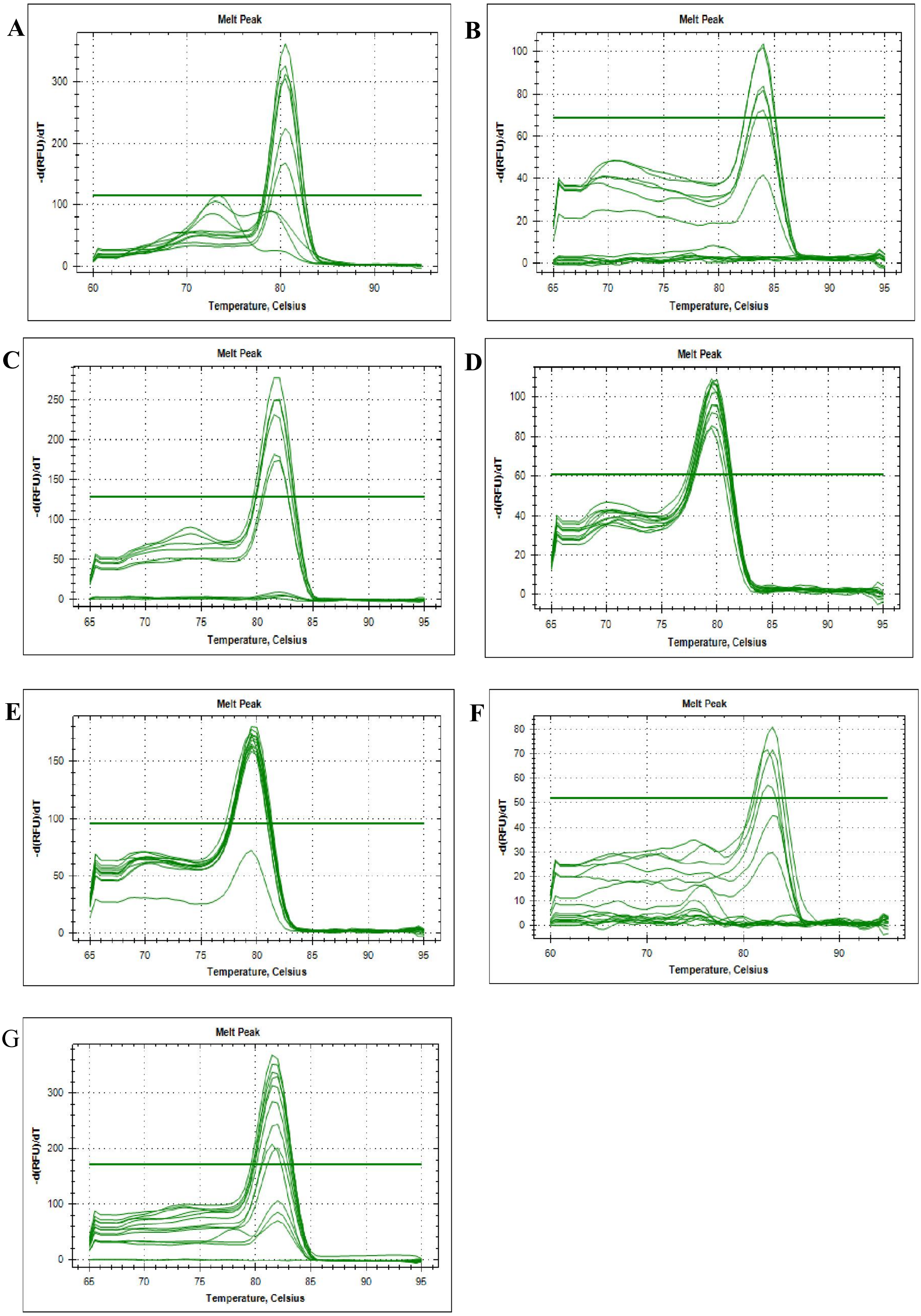

Figure S3. Melting curve analysis of A) *OsLEA1,* B) *OsLEA2,*C) *OsLEA3,* D) *OsLEA4,* E) *OsLEA5,* F) *ACT11* and G) *UBQ5* genes.
